# The association between school class composition and suicidal ideation in late adolescence: Findings from the Young-HUNT 3 study

**DOI:** 10.1186/1753-2000-6-37

**Published:** 2012-11-27

**Authors:** Joakim D Dalen

**Affiliations:** 1 NTNU Social Research, Trondheim, Norway; 2 Department of Sociology and Political science, Norwegian University of Science and Technology, Trondheim, Norway

**Keywords:** Suicidal ideation, Adolescence, School class, HUNT study, Multilevel analyses

## Abstract

**Background:**

Few studies have explored the association between social context and suicidal ideation using multilevel models. This study examines how suicidal ideation in adolescence is related to school class composition.

**Methods:**

Data were obtained from the Young-HUNT 3 study (2006–2008), a population study of adolescents attending secondary school in the Norwegian county of Nord-Trøndelag. The final sample included 2923 adolescents distributed among 379 school classes in 13 schools. Multilevel logistic regression was used to estimate the contribution of various factors at the individual and school class levels.

**Results:**

The results indicate that 5.3 percent of the variation in suicidal ideation can be attributed to differences between school classes. However, a substantial part of this variation can be explained by an unequal distribution of students at risk as a result of individual factors. After controlling for individual-level variables, the results show a higher probability of suicidal ideation in school classes having higher proportions of girls as well as in those following a vocational education programme.

**Conclusion:**

Targeting classes that either follow a vocational education programme or have a high proportion of girls can be an effective approach to intervention because such classes may include a greater number of students at risk for having suicidal thoughts compared to classes with a high proportion of boys or classes following a general education programme.

## Introduction

Suicidal ideation can be defined as “thoughts of engaging in behaviour intended to end one’s life” [1] and is an important indicator of both mental health vulnerability and the risk of engaging in suicide attempts [2,3]. It is especially common during adolescence, with prevalence increasing from age 12 and peaking by age 16, remaining elevated into the early twenties [1].

School classrooms represent an important social context for adolescents. Here, students spend a large portion of their waking hours with a group of classmates who they had no opportunity of choosing themselves and who they are required to interact with [4]. The continuous interaction among the students in each class creates unique psychosocial environments which vary in factors such as shared beliefs, emotions, habits and peer pressure [4,5]. These environments can influence the mental health of students in both positive and negative ways [5]. As a consequence, some school classes are likely to have more students with suicidal ideation compared to others.

It has also been suggested that suicidal ideation may cluster within schools due to suicidal behaviour transferring between individuals as a result of interpersonal interactions with other students who are suicidal [6]. That is, the probability of suicidal ideation could be higher in contexts where there are students with thoughts of taking their own lives who then communicated this ideation outward. If this is the case, then it follows that students who originally are at a low risk for experiencing suicidal ideation may be at higher risk if they have extensive contact with such at-risk individuals.

Multilevel analyses are particularly effective in examining the importance of the school class context because they enable the variation between individuals and groups to be assessed separately [7]. However, multilevel studies investigating the relationship between school context and suicidal ideation are rare [6,8,9]. In the only known study reporting between-school variation in suicidal behaviour, Young et al. [9] found that a small percentage of the variation in attempted suicide (1%), suicide risk (1.3%) and self-harm (1.6%) could be attributed to the school level. The extent to which suicidal ideation may be related to the school classroom context has not been previously examined through the use of multilevel analyses. Research on other mental health outcomes does, however, suggest that the differences between school classrooms are greater than the differences between schools [10-12].

It can be argued that the influence of the social environment on one’s mental health, as well as transference of suicidal ideation, is related to the gender and socioeconomic composition within school classes. Both socioeconomic status and gender are background characteristics often found to be associated with suicidal ideation and mental health. For adolescents, a higher level of parental socioeconomic status is usually associated with fewer mental health problems [13,14], while girls tend to have a higher prevalence of suicidal ideation compared to boys [3,15-19]. If the probability of having suicidal ideation increases as a result of extensive contact with at-risk individuals, then the probability of suicidal ideation should be higher in school classes containing a greater proportion of girls or of students with low socioeconomic background.

Moreover, research has shown that a school’s culture regarding academic achievement can vary greatly depending on the students’ socioeconomic background [5]. Likewise, several studies have suggested that the socioeconomic composition of the school context is associated with mental health status, over and above individual socioeconomic characteristics [6,20-22]. The majority of these studies have found the level of socioeconomic status to be positively related to reports of better mental health, but as with the school context in general, studies specifically examining the relationship between socioeconomic composition of school classes and mental health are scarce. It is, however, likely that school classes, in the same way as schools themselves, will manufacture unique social environments, suggesting that there may be positive effects of a higher average level of socioeconomic background at the class level as well.

Similarly, the influence of one’s psychosocial environment may also depend upon that environment’s gender composition. In a review by Belfi et al. [23], the authors conclude that students in single-sex schools have higher levels of well-being compared to students in mixed schools. This is, however, a gender-specific effect because the relationship has only been documented among girls. Multilevel research analysing the association between classroom gender composition and student mental health is rare, and the few studies testing this relationship have not found significant effects [10].

In this study, suicidal ideation among a population of Norwegian adolescents is examined in relation to school class composition. Suicidal behaviour is a common problem among Norwegian adolescents, and studies on suicidal attempts and self-harm have reported prevalence rates ranging from 3.0 to 8.2 percent [24]. An additional study examining Norwegian conscripts reported a 21.7 percent prevalence rate of life-time suicidal ideation [25], while a second study of adolescents in their last year of upper secondary education (18–19 years) found the prevalence of individuals having suicidal ideation during the last week to be 10.9 percent [26].

To examine the association between suicidal ideation and school class composition, the following two research questions were formulated:

– To what degree can variation in suicidal ideation be attributed to differences between school classes?

– Is there an association between suicidal ideation and school class composition in regards to student gender and parental education?

## Methods

### Data

Participants were identified from the Young-HUNT 3 study, a study population composed of all adolescents attending secondary school (13–19 years old) in the Norwegian county of Nord-Trøndelag. The survey was conducted between 2006 and 2008, and data were acquired through questionnaires and a subsequent health examination. Questionnaires were completed during a school period; consequently, students that had dropped out of school were excluded. The question concerning suicidal ideation was asked only to students in upper secondary school (16–19 years). All 4357 students attending one of the 13 upper secondary schools of the county were invited to participate. Of these, 3353 responded to the questionnaire resulting in a total response rate of 77 percent. After removing cases due to missing data, the final number of students analysed was 2923 distributed across 379 school classes. Participation was voluntary, and every participant was asked to provide written informed consent. Additional information was obtained by retrieving data on parental education from the central registers of Norway. The study was approved by the Regional Committee for Medical and Health Research Ethics.

### The Norwegian school system

After attending ten years of obligatory school, Norwegian adolescents have the option to continue upper secondary school, choosing between three types of general studies and nine types of vocational studies. Of all Norwegian adolescents, approximately 96 percent start upper secondary school, although a substantial number quit during the three to four years of schooling. A majority (96%) of students attend public upper secondary schools, which are administered at the county level.

### Variables

Suicidal ideation was measured by a single question aiming to capture the occurrence of suicidal ideation during one’s lifetime. The question was formulated as: “Have you had thoughts about taking your own life?” Possible response categories were “Yes” and “No”.

Individual explanatory variables included gender, age, socioeconomic status, living situation and parents’ marital status. Parent education level was used to represent socioeconomic status, and the variable consisted of two categories: “Parents with education at college or university level” and “Parents with education lower than college or university level”. When information on both parents’ education was available, the higher level of education was used. If information was not available for both parents, the educational level of the remaining parent was used instead. For the living situation variable, adolescents were grouped based on whether they lived with “both parents”, “one (or mainly one) parent”, “away from home (either alone or with friends)” or “other possible living situations”.

Descriptors of school class composition included the proportion of parents with higher education as well as the ratio of girls to boys in the class. These variables were constructed by aggregating the individual variables of parental education and gender using information on all students participating in the study. Finally, the analyses included variables indicating educational programme (general or vocational) and school grade.

### Statistical analyses

To examine contextual effects on the dichotomous variable of suicidal ideation, multilevel logistic regression analysis was performed. The main advantages of this model are that it allows for the decomposition of unexplained variance between contexts and individuals, as well as effective inclusion of variables on the contextual level. In this analysis, individuals were grouped within school classes. Denoting the probability of suicidal ideation *π*_*tj*_ = Pr(*y*_*ij*_ = 1), where *i* is the individual within school class *j*, the model can be written as

$$\log\left(\frac{\pi_{\mathrm{tj}}}{1-\pi_{\mathrm{tj}}}\right)=\beta_{0}+\beta x_{\mathrm{ij}}+\beta z_{j}+u_{j}$$

*β*_0_ is the intercept, and *βx*_*ij*_ is the vector for the coefficients and values of the variables on the individual level. *βz*_*j*_ is the vector for the coefficients and values on the school class level. Finally, *u*_*j*_ denotes the random effect on the school class level. This random effect is assumed to follow a normal distribution *u*_*j*_ ∼ *N*(0, *σ*_*u*_^2^), with *σ*_*u*_^2^ as the variance parameter of the residual between-school class variance. Using MLwiN, all models were estimated by MCMC methods [27].

## Results

Table 1 presents the descriptive statistics and shows that 22.8 percent of the adolescents in the study reported suicidal ideation. Girls were more likely to report suicidal ideation compared to boys (p<0.001), while adolescents whose parents had a higher level of education were less likely to report suicidal ideation compared to adolescents whose parents had a lower level of education (p<0.05). A higher prevalence of suicidal ideation was also reported among students with divorced parents and among students who were not living with both of their parents (p<0.001).

**Table 1:** Descriptive statistics

**Individual variables**		**N**	**%**	**(%) with suicidal ideation**
Suicidal ideation				
	Yes	668	22.8	
	No	2255	77.2	
Gender				
	Boys	1404	48.0	20.2
	Girls	1519	52.0	25.3
Age				
	16	780	26.7	22.8
	17	1085	37.1	24.3
	18	879	30.1	20.7
	19	179	6.1	23.3
Parental education				
	Less than college or university	1588	54.3	24.5
	College or university	1335	45.7	20.8
School grade				
	First grade	914	31.3	25.5
	Second grade	1195	40.9	25.3
	Third grade	814	27.9	16.3
Living situation				
	Both parents	1749	59.8	18.6
	One parent	643	22.0	28.7
	Away from home	417	14.3	27.6
	Other	114	3.9	37.7
Divorced parents				
	Yes	839	28.7	29.5
	No	2084	71.3	20.2
Educational programme				
	Vocational programme	1366	46.7	28.0
	General programme	1557	53.3	18.4
	Total	2923		22.8
**School class variables**		**N**	**%**	**Mean (std. dev.)**
Educational programme				
	Vocational programme	241	63.6	
	General programme	138	36.4	
Proportion of parents with higher education				0.40 (0.27)
Proportion of girls				0.52 (0.35)
School grade				
	First grade	133	35.1	
	Second grade	166	43.8	
	Third grade	80	21.1	
Total		379		

The prevalence of suicidal ideation was 9.6 percent higher among students enrolled in vocational programmes than those in general programmes. Of the 379 school classes, 241 (63.6%) followed a vocational programme. However, these classes accounted for only 46.6 percent of the students, indicating that classes following a vocational program, on average, were smaller than those in a general educational programme. Finally, data from the table suggest that suicidal ideation was far less common among students in the third grade of upper secondary education.

In multilevel models, a model with no explanatory variables can be used to estimate the intra-class correlation coefficient (ICC) [28]. The ICC is interpreted as the proportion of variance that can be attributed to the higher level in the analysis. Therefore, the ICC provides information of the degree to which suicidal ideation clusters within school classes. In the empty model (not shown) the ICC was estimated to be 0.053 indicating that 5.3 percent of the variance in suicidal ideation could be attributed to the school class level.

Model 1 in Table 2 includes explanatory variables at the individual level. After controlling for other variables, the analyses revealed that boys were less likely to disclose suicidal ideation compared to girls, while parental education and age were negligible factors. In this model, the ICC has been reduced to 2.7 percent, indicating that approximately half of the variance at the school class level could be explained by an unequal distribution of the individual level variables included in the analysis.

**Table 2:** Multilevel logistic regression of adolescent suicidal ideation

		**Model 1**	**Model 2**
**Individual-level variables**			
Gender (ref.: boys)		1.28 (1.07-1.54)**	1.10 (0.88-1.37)
Age (ref.: 16)			
	17	1.05 (0.83-1.33)	1.30 (0.97-1.75)
	18	0.88 (0.68-1.14)	1.77 (1.22-2.57)**
	19	0.89 (0.59-1.34)	1.85 (1.13-3.05)*
Parent education (ref.: low)		0.88 (0.68-1.14)	0.99 (0.80-1.21)
Living arrangement (ref.: both parents)			
	One parent	1.40 (1.01-1.91)*	1.41 (1.03-1.94)*
	Away from home	1.43 (1.08-1.89)**	1.42 (1.07-1.87)*
	Other	2.45 (1.61-3.72)***	2.38 (1.56-3.62)***
Divorced parents (ref.: not divorced)		1.24 (0.94-1.65)	1.20 (0.91-1.58)
**School class-level variables**			
Vocational subject			1.46 (1.16-1.84)**
Proportion of parents with higher education			1.07 (0.65-1.84)
Proportion of girls			1.93 (1.33-2.79)***
School grade (ref.: First grade)			
	Second grade		0.77 (0.58-1.02)
	Third grade		0.37 (0.25-0.54)***
Variance level 2 (Std. Error)		0.11 (0.06)	0.02 (0.030)
ICC (%)		3.2	0.6
Deviance		3088.1	3043.7

*: P < .05; **: P < .01; ***: P < 0.001. OR (95% CI).

In Model 2, variables at the school class level were introduced. Although the proportion of parents with a high educational level had little effect on suicidal ideation, results overall suggested substantial effects of grade, educational programme and gender. Specifically, the effect of school grade indicates that third graders had the lowest probability of suicidal ideation. Regarding educational programmes, the likelihood of having suicidal ideation was substantially higher for those attending a vocational programme compared to those following a general programme. For gender composition, the results indicated that the probability of having suicidal ideation was greater in classes having a higher proportion of girls, even after taking the individual effect of gender into account. It is interesting to note that when the school class level variables were included in the analyses, the individual effect of gender decreased to the point of non-significance.

**Figure 1:**
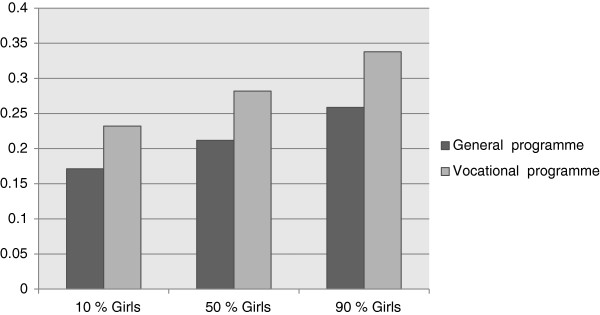
Predicted probability of suicidal ideation by gender composition and educational programme.

Figure 1 shows the predicted probabilities of suicidal ideation by gender balance and type of educational programme. The figure indicates that when all other explanatory variables were held constant, the differences in the probability of having suicidal ideation were substantial. Students in classes following a general educational programme had an approximately 6–8 percent higher probability of suicidal ideation compared to classes that followed a vocational programme. However, the difference is most clearly illustrated when comparing individuals from vocational classes having a large proportion of girls to those in general programme having mostly boys. The probability of suicidal ideation was almost twice as high for the first group compared to the latter.

It is possible that the effects of both individual and contextual variables differ between boys and girls. To examine this possibility, the same models were analysed while stratifying by gender. In this instance, the effects were similar for both genders, and differences were not significant for any of the variables.

## Discussion

The results of this study indicate that a significant amount of variation in suicidal ideation can be attributed to differences between school classes. However, after controlling for individual level variables, it is clear that the unequal distribution of students at risk due to individual factors explained a substantial amount of the variability. Furthermore, the results suggest that the probability of suicidal ideation is higher in classes having a greater proportion of girls as well as in classes following a vocational education programme.

The effect of gender composition on suicidal ideation is surprising as similar effects have not previously been observed. One possible explanation is that in classes with higher proportions of girls, the likelihood of having contact with others with suicidal thoughts is greater due to the overall higher risk of suicidal ideation among girls. Girls also have a tendency to prefer close emotional communication, intimacy and responsiveness in their social relationships [29]. This type of relationship may increase the chance of discussing psychological problems, such as suicidal thoughts, thus increasing the risk of transferring suicidal behaviour. Because school classes with a majority of girls will necessarily have more social relationships that include girls, it is possible that students within these classes will have more suicidal thoughts as a result.

However, the effect of gender composition could also be at least partially explained by mediating or confounding factors not included in these models. At the contextual level, one possibility is that classes with a high proportion of girls create psychosocial environments that can increase the risk for suicidal ideation in some students. On the other hand, perhaps an unequal distribution of individual level variables not included in the model also account for the gender effect. For example, school-related problems such as being bullied, social exclusion, academic stress and academic achievement have all been found to be associated with suicidal ideation [30-32]. If such problems were unequally distributed according to gender composition in our sample, the inclusion of these variables could then remove or reduce the observed effect. Finally, the individual effect of gender disappears when controlling for gender composition, suggesting that the gender differences in suicidal ideation previously observed in the literature [15-19] may partially be explained by the social context.

The absence of an association between aggregated parental education and suicidal ideation is surprising due to the regularity with which this relationship is found in the literature on mental health [13,14]. However, it is possible that the difference is due to limited variation in the population analysed. Nord-Trøndelag is one of the most homogeneous counties in Norway. Compared to other more heterogeneous populations, parental education correlates to a lesser degree with other variables related to socioeconomic status, such as income and neighbourhood disadvantage. This potential explanation is further strengthened by the fact that the vast majority of upper secondary schools are public and free to attend. Consequently, school classes in Norway are much less likely to vary in terms of socioeconomic status compared to societies where school choice is much more dependent on parental socioeconomic background.

Similar results showing that educational programme is significantly associated with suicidal ideation have been observed in the literature on other measurements of mental health [10]. It is possible that the social climates associated with vocational classes differ from classes following a general programme, and thus, may influence the probability of developing suicidal thoughts. Another plausible explanation is that there is a selection effect due to factors associated with both suicidal ideation and choice of school programme. For example, the most important factor in predicting choice of one’s educational programme in Norway is one’s academic achievement during the last year of lower secondary education [33]. As academic achievement has been related to the risk of suicidal ideation [34,35], it is possible that adolescents with suicidal ideation in early adolescence are more likely to choose vocational education programmes. It may also be that this selection effect extends to the gender composition effect. While general education programmes are relatively equal in regards to gender distribution, many of the vocational programmes are not. It is thus possible that the observed effect of gender composition is a result of selection into specific vocational education programmes and not specifically of the gender composition.

That third graders were found to have a lower probability of suicidal ideation was somewhat surprising due to the dependent variable being lifetime suicidal ideation. However, this effect was likely a consequence of the higher probability of suicidal ideation among students dropping out of school [36] as well as individuals having a tendency to forget they had suicidal thoughts [37]. Because the prevalence of suicidal ideation peaks by age of 16 [1], third graders may be more likely to have forgotten their previous suicidal thoughts when compared to first graders.

### Limitations

The unclear causal relationship between school class variables and suicidal ideation is one of the major limitations of the study. This limitation was exacerbated by how the question addressing suicidal ideation was formulated. For instance, the question did not ask specifically when individuals had considered taking their own lives and could therefore have been interpreted by some students as meaning any suicidal ideation in one’s lifetime. Consequently, adolescents may have reported suicidal thoughts that occurred before they began upper secondary education. Furthermore, misreporting of suicidal ideation may have occurred, whether accidental, due to recall, or purposely, due to a lack of anonymity. To minimise this problem, participants were assured that no one at their schools would see their questionnaires. However, such assurances cannot fully guarantee accurate reporting.

Another potential limitation is that the suicidal ideation variable did not take into account the severity of the suicidal thoughts. Adolescents who have thought about taking their own life do not necessarily have mental health problems or suicidal plans. The analyses may have yield different results if suicidal ideation was measured in a way that took severity into account.

Finally, variables on the individual level were restricted to background variables. Including additional individual level variables could alter the observed effects of school class variables, as well as explain unexplained variation at the school class level.

## Conclusion

One of the main reasons for studying suicidal ideation from a school class perspective is that this context may be ideal for interventions. The results of this study indicate that adolescent suicidal ideation is associated with both gender balance and educational programme. Thus, targeting classes with these characteristics may be an effective approach as more students with suicidal ideation are likely to be included in the intervention.

## Competing interests

The author declares that he has no competing interest.

## Authors' contributions

The data were collected as a part of the Nord-Trøndelag Health Study (HUNT) by the HUNT Research Center. JDD did the analyses, interpreted the data and wrote the paper.
